# The Impact of Physical Exercise on the Circulating Levels of BDNF and NT 4/5: A Review

**DOI:** 10.3390/ijms22168814

**Published:** 2021-08-16

**Authors:** Daniel Ribeiro, Luca Petrigna, Frederico C. Pereira, Antonella Muscella, Antonino Bianco, Paula Tavares

**Affiliations:** 1 University of Coimbra, Faculty of Sport Sciences and Physical Education, Coimbra Institute for Clinical and Biomedical Research, 3004-504 Coimbra, Portugal; danieldias577@hotmail.com (D.R.); tavaresc.paula@gmail.com (P.T.); 2 University of Coimbra, Faculty of Medicine, Institute of Pharmacology and Experimental Therapeutics, 3004-504 Coimbra, Portugal; fredcp@ci.uc.pt; 3 University of Coimbra, Faculty of Medicine, Coimbra Institute for Clinical and Biomedical Research, 3004-504 Coimbra, Portugal; 4 Sport and Exercise Sciences Research Unit, Department of Psychology, Educational Science and Human Movement, University of Palermo, 90144 Palermo, Italy; antonino.bianco@unipa.it; 5 University of Coimbra, Center for Innovative Biomedicine and Biotechnology (CIBB), 3004-504 Coimbra, Portugal; 6 Department of Biological and Environmental Science and Technologies (DiSTeBA), University of Salento, 73100 Lecce, Italy; antonella.muscella@unisalento.it

**Keywords:** exercise, neurotrophins, brain-derived neurotrophic factor, neurotrophin-4, peripheral circulation

## Abstract

(1) Background: One mechanism through which physical activity (PA) provides benefits is by triggering activity at a molecular level, where neurotrophins (NTs) are known to play an important role. However, the expression of the circulating levels of neurotrophic factors, brain-derived neurotrophic factor (BDNF) and neurotrophin-4 (NT-4/5), in response to exercise, is not fully understood. Therefore, the aim was to provide an updated overview on the neurotrophin (NT) variation levels of BDNF and NT-4/5 as a consequence of a long-term aerobic exercise intervention, and to understand and describe whether the upregulation of circulating NT levels is a result of neurotrophic factors produced and released from the brain, and/or from neurotrophic secreting peripheral organs. (2) Methods: The articles were collected from PubMed, SPORTDiscus, Web of Science, MEDLINE, and Embase. Data were analyzed through a narrative synthesis. (3) Results: 30 articles studied humans who performed training protocols that ranged from 4 to 48 weeks; 22 articles studied rodents with an intervention period that ranged from 4 to 64 weeks. (4) Conclusions: There is no unanimity between the upregulation of BDNF in humans; conversely, concerning both BDNF and NT-4/5 in animal models, the results are heterogeneous. Whilst BDNF upregulation appears to be in relative agreement, NT-4/5 seems to display contradictory and inconsistent conclusions.

## 1. Introduction

There is growing evidence that insufficient physical activity (PA), highly linked with society’s modern-day sedentary lifestyle, is a major contributor to the increased risk of various health-related problems [1]. On the other hand, it has been well established that PA provides various stimuli that are capable of enhancing both the metabolic and functional status of the human body [2]. Regular PA has evidenced an improvement of the physiological performance of the skeletal and cardiac muscles and, in addition, the decrease of the incidence of a wide range of diseases, including multiple types of cancers, type 2 diabetes, osteoporosis, and brain disease [3]. PA has particularly been known to provoke a cascade of molecular and cellular processes that naturally support brain plasticity [4]. One central mechanism which accounts for the modifications that occur in brain morphology and plasticity is through PA and its ability to produce, release, and regulate growth factors that influence the development of structural and functional changes on the brain [5].

Growth factors are proteins that control various aspects of cellular function, involving survival, proliferation, migration, and differentiation [6]. Subsequently, when considered in the context of the nervous system, the formerly cited growth factors are regularly referred to as neurotrophic factors [7]. To date, four members have been identified as belonging to the mammalian neurotrophic family; nerve growth factor (NGF), brain-derived neurotrophic factor (BDNF), neurotrophin-3 (NT-3), and neurotrophin-4, also referred to as neurotrophin-4/5 (NT-4/5) [8]. Each of these neurotrophic factors bind to one or more cellular receptors and mediate their physiological functions [9]. 

BDNF, one of the most widely reviewed neurotrophic factors belonging to the mammalian family, and NT-4/5, bind to the tyrosine kinase B receptor (trkB) [10]. Moreover, since both bind to the same receptor, it is thought that their roles may be, to a certain extent, similar [11]. BDNF and NT-4/5 also share more than 50% of amino acid homology [12].

Originally, neurotrophins (NTs) were characterized and limited to their role in initial development, growth, maintenance, and the plasticity of the nervous system throughout development, however, current up-to-date literature has indicated the complex role they fulfill during normal physiology in numerous cell populations across both central and peripheral nervous systems, as well as in neuronal and non-neuronal tissues [10,13,14,15]. Although literature in this respect has been fairly inconsistent, recent reports on human and animal models have attempted to examine the impact and link between PA and the upregulation of neurotrophic factor expression and concentrations in multiple body compartments such as the brain, blood, and muscle. Among the numerous well-known exercise training interventions developed throughout these past years, there seems to be a body of evidence reporting that aerobic exercise appears to increase the expression of NTs, particularly BDNF [9,16,17,18]. Contrarily, less clear, and a rather limited number of studies have evaluated the influence of PA on other NTs, such as NT-4/5 [9]. To date, the evidence seems to suggest that there is no sort of unanimity within the relationship between PA, more specifically aerobic exercise, and a consequential NT-4/5 upregulation within compartments. 

One of the main reasons related to the inconsistencies between neurotrophic factor upregulation results and PA is mainly due to discrepancies linked with the heterogeneity between participants characteristics (age, health, status, diseases, etc.), study duration, different intervention exercise programs [4,19], as well as specific methodological considerations, such as blood processing conditions (plasma, serum or whole blood) and timing of collection [4,20], among others. As aforementioned, taken into account that both BDNF and NT-4/5 initiate their intracellular signaling via identical cell surface receptors, it is of interest the reason NT-4/5 has not been evaluated following similar exercise conditions when compared with its neurotrophic counterpart, BDNF [21]. Secondly, according to Pan and colleagues (1998) [22], most members of the mammalian NT family, including BDNF and NT-4/5, appear to cross the blood-brain barrier (BBB), through saturable transport systems, to arrive intact in the central nervous system (CNS), suggesting the possible existence of an exercise-induced neurotrophic crosstalk [23]. Consequently, this increases the difficulty to identify whether in human and animal models these neurotrophic factor fluctuations in serum levels result from changes in central or peripheral tissues. Therefore, the current review aims to provide an updated overview of the NT variation levels of both BDNF and NT-4/5 as a consequence of a long-term aerobic exercise intervention. We will also discuss whether the upregulation of circulating NT levels is a result of the neurotrophic factors produced and released from the brain (cerebral circulation) or, under other conditions, from peripheral organs (peripheral circulation).

## 2. Methods

The manuscript was authored in agreement with the principles of the Preferred Reporting Items for Systematic Reviews and Meta-Analyses (PRISMA) statement [24], having partially adopted the checklist. The protocol was not pre-registered, but was written and approved by the authors before the collection process. Multiple electronic databases were systematically searched from the commencement until 7 July 2020, correspondingly identifying articles published before this date (no restriction was placed on how far back the articles were published). The electronic databases PubMed, SPORTDiscus, Web of Science, MEDLINE, and Embase were used. The following search-term categories were used independently as well as in combination: neurotrophins (e.g., BDNF and neurotrophin-4/5), exercise (e.g., aerobic exercise, treadmill, cycling, swimming), and circulation (e.g., cerebral and peripheral circulation). 

The Population, Intervention, Comparator, Outcomes, and Study design (PICOS) framework design was adopted to create the eligibility criteria for the inclusion of articles in this review. This review included adults (>18 years) and animal models with no restrictions regarding physical or cognitive conditions. Therefore, studies involving individuals with risk factors (e.g., overweight, obesity, diabetes), with known cardiometabolic diseases (e.g., Type 2 diabetes, coronary heart disease), or neurological disorders (e.g., Alzheimer’s disease, depression, mild cognitive impairment) were, too, eligible for inclusion. This review included experimental studies that focused on aerobic exercise intervention models. Any other form of exercise (strength training, HIIT, etc.) was not contemplated in this review. Concerning animal protocols, albeit voluntary wheel-running exercise has been associated in diminishing the potential confounding factor of stress, only forced, involuntary exercise protocols were included as they allow control of the intensity, duration, and timing of the exercise. Restrictions regarding the duration of the intervention were considered. A 4-week training protocol was defined as being the minimum duration-period to be contemplated in this review. Control groups and baseline measurements were considered comparators for intervention effect values. Only original, peer-reviewed, English-language literature was examined. Reviews, meta-analyses, books, book reviews, abstracts, scientific conference abstracts, opinion articles, statements, editorials, letters, and commentaries were excluded.

### Data Collection, Extraction, and Analysis

To identify potentially eligible articles, two investigators independently screened the titles and abstracts acquired from the retrieved articles through the electronic search according to the inclusion and exclusion criteria. Subsequently, after this first selection, full-length articles were read by the same investigators to define which articles consequently met all the inclusion criteria. Nonetheless, in the case of hypothetical inconsistencies or disagreements concerning inclusion or data extraction, a third independent investigator was consulted. Investigators were not blinded to the titles, authors, institutions or manuscript content during the selection process.

Within each of the identified studies, multiple variables were evaluated to determine if they deliberated the association between an aerobic exercise intervention and the changes in BDNF and NT-4/5 levels from pre-exercise to post-exercise. A standardized electronic data extraction method form was developed to obtain information pertinent to this review. Data withdrawal for each article, extracted into the preformatted spreadsheet by two raters, include information regarding: population studied (exercise group vs. control group), sample size, BDNF & NT-4/5 measurement technique used (analysis technique), time of measure, duration, type, and intensity of exercise, as well as the main outcomes. The last item—main outcomes—involves the variation levels of BDNF and NT-4/5 concentrations (increased, decreased, or no change) between pre- and post-exercise intervention values. A subset analysis has also been conducted examining the effects of interventions of less than 12 weeks, between 12 and 36 weeks, and more than 36 weeks. Furthermore, an analysis based on the exercise type and population of the study has also been performed. Data were analyzed through a narrative synthesis.

## 3. Results

**Figure 1:**
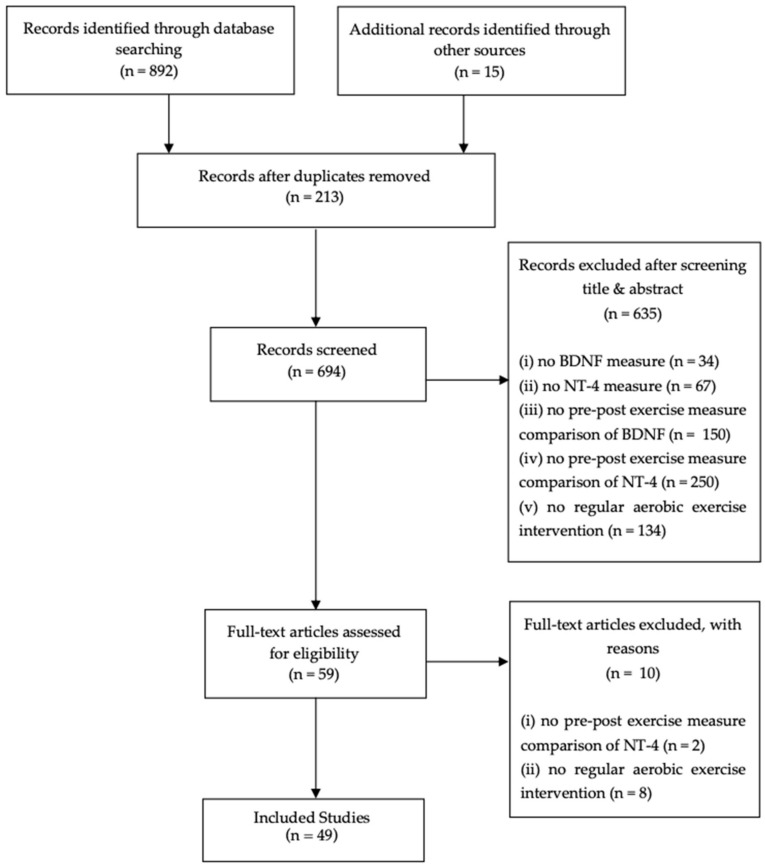
PRISMA (Preferred Reporting Items for Systematic Reviews and Meta-Analyses) flow chart.

An initial raw screening resulted in a selection of 907 articles. Following the titles, abstracts, and a full-text article selection, based on specific predetermined criteria, our search resulted in a final selection of 49 reports (Figure 1).

Regarding the relationship between chronic aerobic exercise interventions and the variation of BDNF blood concentration levels on human subjects, 30 human studies (Table 1) were detected, with a heterogeneous study population varying from young individuals to elderly groups; as well as from healthy study populations to groups with a wide spectrum of medical conditions (e.g., multiple sclerosis [MS], Parkinson’s and Alzheimer’s disease, major depressive disorder [MDD], obese and overweight individuals, etc.). The duration of the exercise protocols varied from 4- to 48-week exercise intervention programs. A wide repertoire of exercise modalities were observed between reports, varying from swimming, treadmill running, cycling, rowing, etc., with the intensity of exercise being relatively similar between the below-mentioned screened articles (moderate-to-vigorous aerobic intensity varying from ±50–80% of VO2max). Most of the analyzed reports (*n* = 24) assessed BDNF measurements from serum samples, whilst some (*n* = 8) used plasma as an alternative measure, and one study, alone, adopted platelet BDNF as an assessment sample. Between all 30 studies conducted on human subjects, the conclusions and main-outcomes varied considerably; within the 30 analyzed, 33 group comparators were established. Of these, 12 in total, which represent ≈36.4% of the entire scope of the review, determined no change whatsoever on the BDNF serum concentration levels post-exercise intervention, when compared with the pre-exercise BDNF concentration values and with the controls; a total of 17 comparators, which represent ≈51.5% of the whole scope of studies, concluded an increase in BDNF concentration levels from pre- to post-exercise intervention; lastly, four comparators determined a decrease in BDNF levels, representing ≈12.1% of the total scope of reviewed articles in Table 1.

### 3.1. Relationship between a Chronic Aerobic Exercise Intervention and the Variation of BDNF Blood Concentration Levels in Human Models

The majority (*n* = 18) of the studies adopted a protocol of fewer than 12 weeks. An increase in BDNF concentration levels was noted after the intervention in ten studies, whilst 4 studies did not detect any variation as a result of an aerobic exercise program. Some studies (*n* = 4) reported contradictory results. Concerning the 10 studies with an intervention period ranging from 12 to 36 weeks, 6 of those studies reported no change whatsoever, three an increase, and one contradictory results regarding BDNF concentration levels. From the two studies of more than 36 weeks, one showed an increase and one a decrease in the BDNF concentration levels after the exercise intervention.

From the twelve studies which proposed a PA intervention in healthy people, seven presented no change in BDNF concentration levels, while a total of four studies cited an increase, and one study reported a decrease in BDNF levels. In four studies concerning people with cognitive impairments, one showed an increase in BDNF concentration levels after a PA intervention, two studies presented no change whatsoever, and one displayed contradictory results. Interestingly, the three studies on overweight and obese people reported an increase in BDNF concentration levels after a PA intervention. The results of PA interventions in schizophrenia or related disorders also seem to be positive, with an increase of BDNF concentrations (*n* = 1). In depressed patients, two studies reported an increase, whilst one study reported no change in BDNF levels after a PA intervention. Studies concerning people with metabolic syndrome (*n* = 2) or type 2 diabetes (*n* = 1) recorded no effects or decreases in BDNF concentrations after the intervention.

Training protocols on either a treadmill or free-running increased BDNF concentration levels in six studies. The same number of studies reported no change after the intervention. A decreased level in BDNF concentrations was reported in one study and contradictory results were observed in three studies.

Alternately, on the cycle ergometer, an increase was observed in nine studies, while no effect of training was seen in seven studies which adopted this type of PA intervention. The results in two studies were conflicting. The only study with rowing ergometer training presented no change in BDNF levels. Results are summarized in Table 1.

### 3.2. Relationship between a Chronic Aerobic Exercise Intervention and the Variation of BDNF Blood Concentration Levels in Animal Models

Concerning the relationship between chronic aerobic exercise interventions and the variation of BDNF blood concentration levels in animal models, 20 studies in total, which met with the exclusion criteria, were extracted (Table 2). The duration between reports was heterogeneous, with the time intervals ranging from 4- to 64-week protocols; the type of exercise varied between swimming and treadmill running; the intensity was considered similar between the articles exhibited in Table 2 (moderate-to-vigorous aerobic intensity). The enzyme-linked immunosorbent assay (ELISA) was the most used analysis technique (*n* = 14); other BDNF measures used were the Western Blot (*n* = 5) and the fluorescence-based real-time PCR quantification method (*n* = 1). The studies (*n*= 20) displayed in Table 2, regarding animal models, suggest that there seems to be a more consolidated agreement amongst authors concerning the positive relationship between regular exercise training and a consequent increase in BDNF production. Of the 20 articles included in Table 2, 18 denote an upregulation in BDNF concentration levels following an exercise protocol, whereas 2 suggest no change whatsoever.

### 3.3. Relationship between a Chronic Aerobic Exercise Intervention and the Variation of NT-4/5 Blood Concentration Levels in Animal Models

Differently from the studies regarding BDNF and long-term aerobic exercise protocols, which consisted of study populations varying from human to animal models, the NT-4/5 studies concerning the relationship between aerobic exercise and the potential NT-4/5 variation in circulation levels, both centrally and peripherally, are only on animal models. As to the relationship between chronic aerobic exercise interventions and the variation of NT-4/5 blood concentration levels in animal models, two studies met our exclusion criteria (Table 3). The minimum duration of the protocol intervention was, as previously stated, of 4 weeks. The exercise modalities between reports were homologous, with treadmill running being the preferred exercise modality amongst both authors’ studies. Again, the intensity of the exercise between reports was analogous, having been considered as moderate. The conclusions differ between reports; if on the one hand, one report suggests that a long-term aerobic exercise protocol promotes a NT-4/5 expression upregulation, the other appears to indicate that NT-4/5 showed no response whatsoever to exercise (Table 3).

## 4. Discussion

NTs, a family of closely related homodimeric polypeptide growth factors, were originally identified for their role in the survival, development, and function of neurons in both central and peripheral nervous systems [73]. The discovery of nerve growth factor (NGF) by Rita Levi di Montalcini and Viktor Hamburger, in the 1950s, represents a breakthrough in the processes that led to modern-day cell biology [74]. After the discovery of NGF and the understanding of the roles it plays in the central and peripheral nervous systems, other neuron promoting neurotrophic factors belonging to the mammalian family were identified, including BDNF, NT-3, and NT-4/5 [75]. These members appear to share a great amount of structural and chemical similarities, including more than 50% of sequence homologies in the primary structure, relatively similar molecular weights, and three disulfide bonds that form a cysteine knot [8]. All NTs are synthesized as larger precursors known as proNTs, and undergo proteolytic cleavage to become mature, biologically active, NTs [76]. These neurotrophic factors exert their biological effects through the binding action from two highly distinct receptors; the tyrosine kinase receptor (Trk), and a member of the tumor necrosis factor superfamily known as the P75 neurotrophin receptor (p75^NTR^) [6,75]. Both pro-NTs—the immature neurotrophic factor form—and mature neurotrophins—bind to p75^NTR^; however, the mature proteins have more affinity with the three members of the tropomyosin-related kinase family of receptor tyrosine kinases [77] when compared to p75^NTR^. Hence, mature NGF binds to TrkA, BDNF and NT-4/5 bind to TrkB, and NT-3 binds to TrkC [10]. However, there seems to occur a certain overlap, due to some redundancy in the structures of the formerly cited neurotrophic factor cell surface receptors, therefore, NT-3 can also bind to TrkA and TrkB, although with less affinity when compared with the third Trk receptor counterpart, TrkC [10]. According to Phillips (2017) [78], proneurotrophin binding to p75^NTR^, via activation of a receptor complex composed of p75^NTR^ and sortilin [79], are known to reduce spine complexity and density [80], induce long-term depression [81], promote neuronal cell death [79], and facilitate the resculpting of neuronal circuits [82]. In opposition to the immature neurotrophic binding interaction, mature NTs, which bind to the Trk receptors, induce, according to Phillips (2017) [78], cell survival and differentiation, dendritic spine complexity, long-term potentiation [83,84], synaptic plasticity, and the resculpting of networks [85]. As aforesaid, only mature NTs bind to the Trk receptors, resulting in the activation of three main pathways: Ras, primarily responsible for the control of normal survival and differentiation by activating mitogen-activated protein kinase; phosphatidylinositol 3-kinase, which manages several neuronal functions such as survival and neurite outgrowth through activation of the protein kinase B, commonly known as AKT; and phospholipase C-γ1, which accounts for the control of activity-dependent synaptic plasticity [8]. Similarly to the Trk receptors, p75^NTR^ also activates three major signaling pathways [86]. The first signaling pathway, NF-kappa B, leads to the transcription of multiple genes; the second, through the activation of Jun kinase via p75^NTR^, leads to neuronal apoptosis; and the last major signaling pathway, Rho activity, controls the growth cone motility [86,87]. Regarding neurotrophic factor secretion, these proteins are known to be produced, released and expressed from both neuronal and non-neuronal cell populations across multiple tissue systems [8,13]. Consequently, although these neurotrophic factors have been acknowledged as molecules responsible for neuronal cell maintenance, it has been well established that they also possess a broad repertoire of functions outside the nervous system [13,15]. Therefore, besides the aforementioned role they exert in neurogenesis regulation, neuronal differentiation and survival, neuronal plasticity, and neuronal conduction [88], they also seem to contribute to the promotion of angiogenesis and survival of adult endothelial cells, vascular smooth muscle cells, and cardiomyocytes; participate in the processes of inflammation and immunity interactions; influence lipid metabolism control; and regulate type 2 diabetes mellitus [89].

### 4.1. BDNF

In 1982, BDNF, the second member of the neurotrophic family of neurotrophic factors, was discovered through the purification of a homogenized pig brain, where it was recognized for its role in the growth and survival of sensory neurons [90]. Currently, BDNF has been identified as a neurotrophic factor that supports the proliferation, survival and differentiation of neurons in the peripheral and central nervous systems [91].

Although BDNF has been extensively characterized for the essential role it plays in neuronal survival and development, known to serve as a neurotransmitter modulator, and participate in neuronal plasticity [89,92], it also seems to promote actions on cardiac and endothelial cells [93], act as a mediator between airway inflammatory events and neuronal changes (inflammation and immunity) [89], affect energy metabolism (lipid metabolism) [94,95], is also implicated in the cytoprotective action in type 2 diabetes mellitus [96], and, through the ability of this neurotrophic factor to enhance neurogenesis and consequently improve synaptic plasticity, as formerly discussed, evidence suggests that it may be highly linked with multiple neurological conditions such as Alzheimer’s disease, dementia, Huntington’s disease, bipolar disease, and autism [41,49,50].

As previously mentioned, the cellular processing of neurotrophic factors, especially of BDNF, has been significantly reviewed throughout these past years within the literature [97]. Synthesis and maturation of BDNF consists of a multistage process, involving the sequential formation of numerous precursor isoforms [98]. BDNF is initially synthesized as a precursor, identified as a folded pre-pro-BDNF form in the endoplasmatic reticulum [98,99]. Subsequently, the pre-region sequence is removed upon translocation through the Golgi membrane, yielding the 32 kDa isoform, consisting of 129 amino acid proteins of BDNF, also referred to as pro-BDNF [98,99]. Consequently, following the cleavage of the signal peptide, the pro-BDNF isoform is converted to a mature BDNF isoform (m-BDNF) by intracellular (e.g., PC7, furin, and proconvertases), or by extracellular proteases (e.g., metalloproteinases and plasmin), resulting in a 13 kDa polypeptide [30,54,55]. Accumulating evidence has suggested that contrary to what was previously thought, both pro-BDNF and its mature isoform, m-BDNF, are biologically active [100]. Furthermore, the mature form of BDNF is identical between all mammals [101]. Regarding the interactions with different types of receptors according to BDNF’s multiple isoforms, pro-BDNF interacts, preferentially, with the p75^NTR^ through its mature domain, and with the sortilin receptor [98], whereas m-BDNF binds with the TrkB receptor [102]. This neurotrophic factor is found in elevated concentrations in the CNS, predominantly in the brain regions of the hippocampus, cerebral cortex, hypothalamus, and cerebellum [103]. Central BDNF can cross the BBB and, therefore, be found in the bloodstream. However, circulating BDNF can be derived from multiple sources other than the CNS [104,105]. Accordingly, it has been well established that a great number of tissues produce and release this neurotrophic substance, including the lungs, bladder, intestinal tissue, vascular endothelial cells, skeletal and cardiac muscle, peripheral neurons, peripheral blood mononuclear cells, and platelets [106,107,108,109,110,111,112,113,114]. Therefore, it is seemingly difficult to ascertain the peripheric and brain contribution to the seric BDNF levels [104].

The source of BDNF in the peripheral circulation raises several questions and is far from being clear. In 2018, Naeglin et al. [115] searched for the range of BDNF levels that could be considered normal in human blood. They found that BDNF can be measured in human plasma, however, comparisons between populations were not reliable, consequently needing future cohort studies. They also found a correlation between platelet and serum BDNF. Nevertheless, the source of plasma/serum and platelet BDNF was not clarified. On the other hand, a work by Chacón-Fernandéz and colleagues (2016) [116] referred that the possible source of platelet BDNF are megakaryocytes, the progenitors of platelets. Moreover, the same authors suggested that the induced-BDNF release by physical exercise may reflect the changes that happen in megakaryocytes and platelets, including the ability of the latter to retain and release BDNF. In spite of these articles, it is known, as referred to previously, that other organs and tissues, such as the skeletal muscle, are able to produce considerable amounts of BDNF during contraction (aerobic physical exercise); however, the skeletal muscles contribution in the variation levels in plasma and brain BDNF remains unclear.

### 4.2. NT-4/5

Although the trophic effects on central and peripheral neurons have been well-established both in vivo and in vitro, NT-4, also referred to as NT-4/5, continues to be the least studied member of the mammalian NT family [117]. According to László, et al. (2019) [118], NT-4/5 is responsible for promoting survival and differentiation of hippocampal, noradrenergic and dopaminergic neurons, as well as being involved in the development of chemo-afferent sensory neurons known to innervate the carotid body; furthermore, this neurotrophic factor has a strong survival and proliferative action on NIH 3T3 cells expressing TrkB, however, it seems to have relatively limited activity on 3T3 cells expressing TrkA [119]. Except for being the most recently isolated neurotrophic factor of the mammalian family, as well as being the least well understood, numerous features seem to characterize this protein in a unique manner, when compared with its mammalian neurotrophic family counterparts [120]. Thus, in contrast to the other NTs, its expression is ubiquitous and appears to be less influenced by environmental signals [120]; in addition, it is also expressed at substantially inferior levels when compared with any other neurotrophic factor [121].

NT-4/5 was initially identified in *xenopus* and *viper*, having later been discovered in both rats and humans [119]. The mature NT-4/5 sequences between human and rat models are 95% identical at an amino-acid level [122]. Consequently, the immature form of both human and rat NT-4/5 isoforms consists, respectively, of a 210 and a 209 amino-acid sequence; following the removal of the prepropeptide, both human and rat NT-4/5, in its mature form, consists of a 130 amino-acid chain [119], with a molar mass equivalent to 14 kDa [123]. Depending on its isoform—mature or immature—NT-4/5 exerts its biological activities by binding to either TrkB or p75^NTR^ [124,125].

In contrast to BDNF, NGF and NT-3, information concerning the sites of synthesis of NT-4/5 are quite limited, with low concentrations being detected in the whole rat embryo, the whole brain of the adult rat and in some rat and human peripheral tissues [122,126]. Concerning NT-4/5 detection within tissues, results revealed by Timmusk and colleagues (1993) [121] show a developmentally regulated expression of NT-4/5 mRNA in most tissues, with an apparent functional implication for NT-4/5 in the nervous system (e.g., ten brain regions including x and y), as well as in peripheral and non-neuronal tissues (e.g., heart, liver, muscle, skin, lung, kidney, thymus, spleen, submandibular gland, pituitary, thyroid, testis, ovary); however, following conclusions presented by previous authors [122,126], results from Timmusk and colleagues (1993) [121] reveal low levels of NT-4/5 mRNA in a wide range of embryonic and adult rat tissues. Tissue distribution of human NT-4/5 transcripts was identified in a limited number of peripheral tissues, with the highest levels found in the prostate and lower levels in the thymus, placenta, skeletal muscle, and the testis; NT-4/5-hybridizing transcripts were not found in the brain [122]. This neurotrophic factor is also present in human serum [127] and is also capable of crossing the BBB [22]. This is suggestive that these NTs can travel both ways: from the CNS to the periphery and from the periphery into the brain. 

### 4.3. Physical Exercise & Neurotrophic Factors

A kaleidoscope of significant benefits of PA, particularly aerobic exercise [9,128], on human health, have long been correlated and predominantly include the decreased risk of cardiovascular disease, diabetes, cancer, osteoporosis, and CNS diseases [9,129]. Only recently, however, has it been admitted that PA may also stimulate a panoply of molecular and cellular processes [130], known to modulate a cascade of chemical messengers referred to as neurotransmitters and NTs, which act in an activity-dependent manner, resulting in the potentiation of the neural function and in the induction of several events which support the structural and functional plasticity of the brain [5,60,86]; nonetheless, the exact mechanisms through which PA induces an upregulation of neurotrophic factor expression have still not been fully elucidated. 

The use of animals for scientific purposes is a relatively antique practice used in biological research and medicine, however, associated with this methodical scientific approach is an array of recurrent ethical concerns [131,132]. There are extraordinary anatomical and physiological similarities between human and animal mammalian models [131] and, as a consequence, an extensive number of reports have been carried out to study and comprehend the relationship between PA and NTs in cellular and animal models [9]; however, it must be taken into consideration that not all of the results can be forthrightly translated to humans [131] and, therefore, must be interpreted with caution.

### 4.4. BDNF & Aerobic Exercise

BDNF is one of the most extensively reviewed neurotrophic factors belonging to the mammalian family. Therefore, it is not surprising that there is a great number of reports focusing and investigating the effects of PA, in particular aerobic exercise, on BDNF’s concentration variation levels in both human and animal models. One major hallmark related with the neurotrophic factor and physical exercise field of study was the discovery of an active interface known as the BBB, which allows the crosstalk of certain substances (e.g., polypeptides, such as NTs and cytokines) between the periphery and the CNS [133].

### 4.5. NT-4/5 and Aerobic Exercise

Contrasting the great number of reports and consequential data available regarding BDNF, a reasonably limited sum of studies have assessed the influence of long-term aerobic exercise protocols on the variation levels of NT-4/5 in the cerebral and peripheral circulation [9]. Peculiarly, NT-4/5 studies concerning the relationship between aerobic exercise and the potential NT-4/5 variation in circulation levels, both cerebrally and peripherally, only regard animal models. The conclusions differ markedly between reports; if on one hand, the main outcome from one study [58] suggests that a long-term aerobic exercise protocol promotes a NT-4/5 expression upregulation, the other [18] appears to suggest that NT-4/5 showed no response whatsoever to exercise.

### 4.6. The Impact of Aerobic Exercise on Circulating Neurotrophin Levels (BDNF and NT-4/5) from Cerebral and Peripheral Circulation: Which Organs Are the Main Contributors?

BDNF, which is present in the blood both at rest and during exercise training is, according to Walsh & Tschakovsky (2018) [105], most likely derived from several tissue sources (lungs, bladder, intestinal tissue, vascular endothelial cells, skeletal and cardiac muscle, peripheral neurons, peripheral blood mononuclear cells, platelets, and the brain) known to produce and release neurotrophic factors into circulation in response to exercise-like stimuli [105,106,107,108,110,113]. As previously mentioned, contrary to BDNF and other members of the neurotrophic factor family, information regarding NT-4/5 is limited. The conclusions presented by Timmusk and colleagues (1993) [121] seem to be in accordance with those also admitted by Ibañez (1996) [120] in the means that this neurotrophic substances expression—NT-4/5—seems to be undoubtedly ubiquitous, found in a broad range of tissues and organs, such as in the heart, liver, muscle, thymus, lung, kidney, testis, ovary, salivary gland, cerebral cortex, brain stem and the hippocampus [121].

As it has been formerly stated, one major hallmark related to the neurotrophic factor and PA field of study was the discovery of the BBB, which has been acknowledged in enabling the crosstalk of NTs—e.g., BDNF and NT-4/5—through saturable transport systems, between the periphery and the CNS [133]. Taken into account that both BDNF and NT-4/5 have been found to be produced and expressed in a variety of tissues and organs, and are correspondingly found in the periphery and CNS, suggesting the existence of a neurotrophic crosstalk loop between systems through the BBB, it is of particular interest to understand up to what extent these multiple cell-populations, known to produce and express neurotrophic factors, contribute in the total amount of BDNF and NT-4/5 found in the blood. 

To date, the only study that seems to evaluate the contribution of the human brain to plasma BDNF at rest and during prolonged whole-body exercise was one published by Rasmussen and colleagues (2009) [134]. According to the previously mentioned author, following a four-hour rowing ergometer protocol, completed by eight volunteers, and a two-hour treadmill session, completed by 32 mice, the main outcomes were that in humans, a BDNF release from the brain was detected at rest (*p* < 0.05), and increased two to threefold during exercise (*p* < 0.05); concerning the mice study group, exercise provoked a three to fivefold upsurge in BDNF mRNA expression in the hippocampus and cortex, peaking 2 h after the conclusion of the exercise protocol. The results obtained by Rasmussen and colleagues (2009) determined that both at rest and during exercise, the brain contributed for up to 70–80% of the circulating BDNF, consequently suggesting that the brain is, in fact, the main, but not the only contributor, of circulating BDNF. On the other hand, in the case of NT-4/5, to date, no study whatsoever has been conducted in the means to understand up to what extent tissues from the CNS and from the periphery contribute to the NT-4/5 levels found in the blood.

This review has limitations. Firstly, the sample characteristics were not always specified by the authors, making it impossible for an accurate subgroup analysis. Furthermore, the physical fitness levels of the participants that took part in the studies were not always detected, making it difficult to evaluate the conclusions of the included studies. Additionally, especially in the animals’ model studies, the inconsistencies in the duration, type and intensity of the exercise interventions, as well as specific methodological considerations such as blood processing conditions (plasma, serum or platelet), contributed to the subsequent variability of results. Further reports, with both increased sample sizes and longer durations, are needed to evaluate the effect of a chronic aerobic exercise intervention on blood BDNF and NT-4/5 concentrations in a more conclusive manner. Moreover, additional research regarding the relationship between peripheral and cerebral concentrations of both BDNF and NT-4/5 is required to evaluate the effect of long-term (chronic) aerobic exercise on the BDNF and NT-4/5 concentration levels in the blood.

## 5. Conclusions

This review provides, firstly, an updated overview on the evidence concerning the role that long-term aerobic exercise programs may induce on BDNF and NT-4/5 blood concentration levels and, secondly, attempted to understand up to what extent the literature studied the contribution of cerebral and peripheral BDNF and NT-4/5 secreting cell-populations, responsible for these NTs found in the blood.

Regarding the relationship between BDNF and long-term aerobic exercise protocols, studies performed in human populations appear to demonstrate a certain level of ambiguity on the effects that PA, more specifically aerobic exercise, provokes in the stimulation and enhancement of BDNF blood concentration levels. On the other hand, in animal models, the BDNF and long-term aerobic exercise protocol relationship appears to be relatively unquestionable, in the means that regular exercise training does, indeed, in most cases, lead to an increase in BDNF concentration levels found in the blood.

Concerning the relationship between NT-4/5 and long-term aerobic exercise protocols, evidence is fairly inconsistent due to the lack of studies, consequently making it difficult to reach a final conclusion.

## Figures and Tables

**Table 1:** Summary of studies that have reviewed the relationship between a chronic aerobic exercise intervention and the variation of BDNF blood concentration levels in human subjects.

Author(s)	Population Studied (Exercise Group *n* vs. Control Group *n* )	*n*	BDNF Measure (Analysis Technique)	Time of Measure	Duration of Exercise	Aerobic Exercise (Days p/Week of Training)	Intensity of Exercise for Exercise Group (Physiological Parameter)	Main Outcomes
**<12 weeks**								
Griffin, et al. (2011) [25]	Young Sedentary Healthy Adult Males (3-week Aerobic Exercise Group-C-Ex3 *n* = 9 vs. 5-week Aerobic Exercise Group C-Ex5 *n* = 9 vs. Sedentary Non-Exercise Control Group-CON *n* = 15)	33	Serum (ELISA)	pre-exercise and post-exercise at weeks 3/5	3 weeks & 5 weeks	stationary cycle ergometer (3 days/week)	60% VO2max	3 weeks of cycling training presented no effect on serum BDNF concentration; however, an increase of serum BDNF concentration was observed at week 5.
Salehi, et al. (2016) [26]	Major Depressive Disorder Patients (Aerobic Exercise Group *n* = 20)	20	Plasma (ELISA)	pre- and post intervention	4 weeks	cycling on a treadmill (3 days/week)	60–75% of VO2max	Pre- to post-assessment plasma BDNF levels were increased in the Aerobic Exercise Training group.
Zoladz, et al. (2008) [27]	Healthy Physically Active Young Men (Exercise Group *n* = 13)	13	Plasma (ELISA)	pre- and post intervention	5 weeks	cycle ergometer (4 days/week)	90% of VO2 at lactacte threshold	A 5-week exercise program of moderate intensity endurance training resulted in a significant “chronic” increase in the basal, as well as in the exercise induced “acute” increase in plasma BDNF levels.
Kerling, et al. (2017) [28]	Depressed Inpatients (Aerobic Exercise Group + Treatment as Usual *n* = 22 vs. Treament as Usual Control Group *n* = 20)	42	Serum (Quantikine Sandwich Enzyme Immunoassay)	pre- and post intervention	6 weeks	bicycle ergometer, treadmill, cross-trainer or rowing (n.d.)	n.d.	An increase in serum BDNF in the exercise group was detected. Alterations over time reached no statistical significance in both groups. The Treatment as Usual Control Group serum BDNF levels decreased slightly. Exercise appears to have additional effects on BDNF serum concentrations in people with major depressive disorder.
Babaei, et al. (2013) [29]	Metabolic Syndrome (MetS) and Healthy Middle-Aged Males (MetS-Exercise *n* = 11 vs. MetS-Control *n* = 10 vs. Healthy-Exercise *n* = 11 vs. Healthy-Control *n* = 10)	40	Serum (ELISA)	pre- and post intervention	6 weeks	walking &/or running (3 days/week)	between 50–60% of VO2peak	After aerobic exercise, BDNF levels significantly increased in the Healthy Exercise Group, but decreased in the Metabolic Syndrome Exercise Group.
Damirchi, et al. (2014) [30]	Middle-Aged Men Diagnosed With Metabolic Syndrome (Aerobic Training Exercise Group *n* = 11 vs. Non-exercise Control Group *n* = 10)	21	Serum (ELISA)	pre- and post intervention	6 weeks	walking or running (3 days/week)	50–60% of VO2 peak	Serum BDNF significantly decreased after chronic aerobic training in the Metabolic Syndrome exercise group subjects (Blood samples were collected at the end of the 6-week training).
Wagner, et al. (2017) [31]	Male Students (Aerobic Exercise Group *n* = 17 vs. Control Group *n* = 17)	34	Serum (ELISA)	pre- and post intervention	6 weeks	bicycle ergometer (3 days/week)	77± 9% of pre-training VO2max (ranging from 60% to 88%)	The results indicate a decrease in the exercise-induced BDNF concentration after the intervention in the exercise group and an increase in the control group. Unchanged baseline BDNF serum concentrations and decreased exercise-induced BDNF levels were reported in the Exercise Group.
Castellano & White, (2008) [32]	Multiple Sclerosis (MS) (MS Exercise Group *n* = 11 vs. Healthy Exercise Control Group *n* = 11)	22	Serum (Quantikine Sandwich Enzyme Immunoassay)	pre- and post intervention	8 weeks	cycle ergometer (3 days/week)	60% VO2peak	Resting serum BDNF was significantly lower in MS compared to control subjects at week 0, but was not significantly different at week 8. In the MS exercise group, an elevated concentration of serum BDNF was observed following 4 weeks of training with a return to baseline at week 8. Non-significant difference in the MS group at 8 weeks. In contrast, resting BDNF concentrations remained unchanged at 4 and 8 weeks of training in controls.
Cho & Roh (2016) [33]	Young Obese Men (Aerobic Exercise Group *n* = 8 vs. Non-Exercise Control Group *n* = 8)	16	Serum (ELISA)	pre- and post intervention	8 weeks	treadmill running (3 days/week)	70% of the heart rate reserve (HRR)	Following the intervention (regular aerobic exercise training), serum BDNF levels were significantly higher than those prior to the intervention in the exercise group of obese individuals.
El-Tamawy, et al. (2014) [34]	Ischemic Stroke Patients (G2 subjected to a physiotherapy program followed by aerobic exercise *n* = 15 vs. G1-control group-subject to a physiotherapy program without aerobic exercise *n* = 15)	30	Serum (ELISA)	pre- and post intervention	8 weeks	bicycle ergometer (3 days/week)	n.d.	Aerobic exercise, following an acute ischemic stroke, is accompanied by an increase in the serum levels of BDNF. At the end of the treatment, compared with the pre-concentration levels, there was a significant increase in the serum BDNF levels within the exercise group, while the control group showed no significant increase in the serum BDNF levels.
Goekint, et al. (2010) [35]	Young Sedentary Students (Exercise Group *n* = 9 vs. Physically Inactive Control Group *n* = 7)	16	Serum (ELISA)	pre- and post intervention	8 weeks	walking, running, cycling, synchro, wave (3 days/week)	n.d.	Peripheral serum BDNF concentration levels were not influenced by an 8 week aerobic training protocol.
Marusiak, et al. (2015) [36]	Mild-to-Moderate Parkinson Disease Patients (Parkinson Disease Patients Exercise Group *n* = 11 vs. Healthy Non-Trained Control Group *n* = 11)	22	Serum (Assay/Microplate Reader)	pre- and post intervention	8 weeks	stationary cycloergometer (3 days/week)	62–68% of HRmax	The BDNF serum levels increased in the Parkinson Disease Patients from the exercise group, whereas no change was observed in the Healthy Control Group. Training resulted in an increase in BDNF levels relative to the pre-training values.
Roh & So (2016) [37]	Obese and Non-Obese Men (Obese Exercise Group *n* = 10 vs. Healthy Non-Obese Exercise Group *n* = 10)	20	Serum (ELISA)	pre- and post intervention	8 weeks	treadmill running (3 days/week)	70% heart rate reserve	The obese group showed a significantly lower BDNF level at baseline compared with the non-obese group. The non-obese group showed no significant difference in blood neurotrophic factor levels before and after training, whereas the obese group showed a significantly higher BDNF level after training.
Schulz, et al. (2004) [38]	Multiple Sclerosis (MS) (Exercise Group *n* = 15 vs. Control-no intervention-Group *n* = 13)	28	Serum (ELISA)	pre- and post intervention	8 weeks	bicycle ergometer (2 days/week)	60% VO2max	Although not with statistical significance, an increase of BDNF levels was seen in the MS training group, while the levels in the MS control group decreased.
Zoladz, et al. (2014) [39]	Patients with Idiopatic Parkinson’s Disease (Exercise Group *n* = 12)	12	Serum (ELISA)	pre- and post intervention	8 weeks	stationary cycle ergometer (3 days/week)	60–75% of HRmax	Serum BDNF levels increased significantly after an 8-week moderate-intensity interval training intervention in the Parkinson Disease Exercise Patients.
Enette, et al. (2020) [40]	Seniors with Mild to Moderate Alzheimer’s Disease (Continuous Aerobic Exercise Group-CAT *n* = 14 vs. vs. Control Group *n* = 21)	35	Plasma (ELISA)	pre- and post intervention at week 10	9 weeks	cycle ergometer (2 days/week)	70% of HRmax	9 weeks of continuous aerobic training failed to induce significant plasma BDNF response compared to baseline levels. No significant change was measured in terms of plasma BDNF levels after the training program.
Briken, et al. (2016) [41]	Patients with Primary or Secondary Progressive Multiple Sclerosis (Exercise Group-arm ergometry/rowing or bicycle ergometry *n* = 28 vs. Control Group *n* = 9)	37	Serum (ELISA)	pre- and post intervention	9 weeks	arm ergometry, rowing or bicycle ergometer (2–3 days/week)	*n*.d.	BDNF serum levels did not increase significantly after a training intervention of 22 sessions in comparison to the control group. Long-term effects of BDNF are less pronounced when compared with acute exercise.
Araya, et al. (2013) [42]	Overweight and Obese subjects (*n* = 15)	15	Serum, Plasma & Platelet (ELISA)	pre- and post intervention	10 weeks	treadmill or bike + stretching (3 days/week)	≥65% VO2max	In sedentary, nondepressed, overweight and obese subjects, serum and platelet BDNF circulating levels increased after 30 sessions of aerobic exercise.
**12–36 weeks**								
Kimhy, et al. (2015) [43]	Schizophrenia or Related Disorder Individuals (Aerobic Exercise Group + Treatment As Usual *n* = 16 vs. Control Treatment As Usual Group *n* = 17)	33	Serum (ELISA)	pre- and post intervention	12 weeks	treadmill, stationary bike or eliptical machine (3 days/week)	60–75% of HRmax	At follow-up, BDNF increased in the Aerobic Exercise group. Group difference changes in BDNF did not reach significance, potentially due to the small sample size.
Krogh, et al. (2014) [44]	Major Depression Patients (Aerobic Exercise Group *n* = 41 vs. Control Group *n* = 38)	79	Serum (ELISA)	pre- and post intervention	12 weeks	stationary bikes (3 days/week)	80% of HRmax	A 3-month aerobic exercise training program did not increase BDNF serum levels. No differences were found in serum BDNF between the aerobic exercise group and the control group in the post-intervention samples.
Maass, et al. (2016) [45]	Healthy Sedentary Older Adults (Aerobic Exercise Group *n* = 21 vs. Control Group *n* = 19)	40	Plasma & Serum (ELISA)	pre- and post intervention	12 weeks	stationary treadmill (3 days/week)	65% of target heart rate +5% in steps for 4 weeks	BDNF did not significantly change between pre- and post exercise levels. The 3-month intervention did not alter BDNF levels in the blood.
Matura, et al. (2017) [46]	Healthy Older Adults (Aerobic Exercise Program Group *n* = 29 vs. Non-Exercise Control Group *n* = 24)	53	Serum (ELISA)	pre- and post intervention	12 weeks	cycle ergometer (3 days/week)	64 ± 9% VO2 max	No effect of aerobic exercise training was seen on serum BDNF concentrations compared with the control group.
Seifart, et al. (2010) [47]	Healthy Sedentary Males (Endurance Training *n* = 7 vs. Sedentary Controls *n* = 5)	12	Plasma (ELISA)	pre- and post intervention	12 weeks	cycling, running, swimming or rowing (7 days/week)	70% of maximal HR, equivalent to approximately 65% VO2max	3 months of endurance training enhanced the resting release of BDNF, with no significant change in the control group. There was no training-induced increase in the release of BDNF during exercise.
Schiffer, et al. (2009) [48]	Healthy Sports Students (Moderate Endurance Training Group vs. Control Group)	27	Plasma (ELISA)	pre- and post intervention	12 weeks	treadmill (3 days/week)	80% of the HR at the aerobic-anaerobic threshold	There were no significant changes for BDNF. The authors suggest that exercise *per se* does not result in changes in plasma concentrations of BDNF.
Williams & Ferris, (2012) [49]	Physically Active & Healthy Young Subjects (*n* = 18)	18	Serum (ELISA)	pre- and post intervention	12 weeks	jogging (3 days/week)	between 65–70% of HRmax	BDNF was unchanged in response to the moderate intensity training program. A 12 week aerobic training program of moderate intensity, completed by healthy subjects, did not result in an increase in BDNF concentrations.
Baker, et al. (2010) [50]	Adults with Amnestic Mild Cognitive Impairment (High-Intensity Aerobic Exercise Group *n* = 19 vs. Stretching Control Group *n* = 10)	29	Plasma (ELISA)	pre- and post intervention	24 weeks	treadmill, stationary bicycle, or elliptical trainer (4 days/week)	75% to 85% of HR reserve	Relative to controls, aerobic exercise increased circulating levels of BDNF in men patients and decreased levels of BDNF in women.
Cho, et al. (2014) [51]	Healthy Middle-Aged Women (Aerobic-Exercise Group *n* = 15 vs. vs. Non-exercise Control Group *n* = 7)	22	Serum (ELISA)	pre- and post intervention	24 weeks	treadmill (4 days/week)	between 50–80% of VO2max	After 24 weeks, there were significant serum BDNF level changes in the aerobic exercise group, when compared to the control group.
Ruscheweyh, et al. (2011) [52]	Older Healthy Individuals (Medium-Intensity Aerobic Exercise Nordic Walking Group *n* = 20 vs. Exercise Gymnastics Low-Intensity Aerobic Group *n* = 21 vs. No Intervention Control Group *n* = 21)	62	Serum (ELISA)	pre- and post intervention	24 weeks	nordic walking & gymnastics (3 days/week)	medium-intensity aerobic exercise: (50–60% of maximal exertion) and low-intensity aerobic exercise (30–40% of maximal exertion)	Change in physical activity levels with the intervention showed a trend for a positive association with the change in BDNF levels. The authors detected a trend of an increase in BDNF with increasing physical activity over six months.
**>36 weeks**								
Swift, et al. (2012) [53]	Type 2 Diabetes Individuals (Aerobic Training Only *n* = 40 vs. Resistance Training Only *n* = 44 vs. Combination of Resistance and Aerobic *n* = 43 vs. Non-exercise Control Group *n* = 23)	150	Serum (Chemkine Sandwich Enzyme Immunoassay)	pre- and post intervention	36 weeks	treadmill (150 min/week)	between 50–80% of VO2max	Serum BDNF measures were not altered by 9 months of aerobic, resistance, or combination (aerobic + resistance) training when compared with the non-exercise control group.
Erickson, et al. (2011) [54]	Older Adults (Moderate-Intensity Aerobic Exercise Group *n* = 60 vs. Stretching Control Group *n* = 60)	120	Serum (Quantikine Sandwich Enzyme Immunoassay)	pre- and post intervention	48 weeks	treadmill (3 days/week)	moderate-intensity 50–60% of HR reserve (1–7 weeks) 60–75% HR reserve (remainder of the program)	The aerobic exercise group did not demonstrate greater changes in circulating serum BDNF levels compared with the control group. However, the aerobic exercise group revealed higher post-intervention BDNF levels, when compared with the pre-intervention BDNF concentrations.

**Table 2:** Summary of studies that have reviewed the relationship between a chronic aerobic exercise intervention and the variation of BDNF blood concentration levels in animal models.

Author(s)	Population Studied (Exercise Group *n* vs. Control Group *n*)	*n*	BDNF Measure (Analysis Technique)	Time of Measure	Duration of Exercise	Aerobic Exercise (Days p/Week of Training)	Intensity of Exercise for Exercise Group (Physiological Parameter)	Main Outcomes
*<12 weeks*								
Ahn, et al. (2016) [55]	Aged Mongolian Gerbil Males (Sham-Operated Group *n* = 14 Ischemia-Operated 1-week Exercise Group *n* = 14 vs. Ischemia-Operated 4-week Exercise Group *n* = 14 vs. 4-week Sedentary Ischemia-Operated Control Group *n* = 14)	56	western blot	post-intervention	1 and 4 weeks	treadmill running (5 days/week)	n.d.	In the Ischemia Operated 4-week Sedentary Group, BDNF protein levels were increased compared with those in the Sham-Operated Group; BDNF protein levels in the Ischemia-Operated 4-week Exercise Group were significantly increased compared with the Ischemia Operated 4-week Sedentary Group, and Ischemia-Operated 1-week Exercise Group. 4 weeks of exercise (long-term exercise) enhanced the BDNF expression.
Liu, et al. (2008) [56]	Male BALB/c Mice (Exercise Group *n* = n.d. vs. Control Group *n* = n.d.)	n.d.	ELISA	post-intervention	4 weeks	treadmill exercise (5 days/week)	n.d.	The exercise effect of training on hippocampal BDNF level was acute rather than chronic; training transiently increased the hippocampal BDNF level at 1, 2, and 4 h post-exercise when compared to the control.
Liu, et al. (2009) [57]	Male BALB/c Mice (Forced Treadmill Exercise Group *n* = n.d. vs. Control Group *n* = n.d.)	n.d.	ELISA	post-intervention	4 weeks	treadmill exercise (5 days/week)	n.d.	Training elevated BDNF levels. Forced treadmill running transiently elevated hippocampal BDNF levels and significantly increased BDNF levels in the amygdala compared to the control.
Maejima, et al. (2018) [18]	Senescence-Accelerated Mice (Control Group *n* = 6 vs. Resistant Exercise Group *n* = 6 vs. Prone Sedentary Control Group *n* = 8 vs. Prone Exercise Group *n*= 7)	27	ELISA	post-intervention	4 weeks	treadmill running (5 days/week)	n.d.	The study revealed that aerobic exercise for 4 weeks incresed transcriptional and protein expression levels of BDNF in mice. Long-term exercise enhances the expression of BDNF in the hippocampus.
Skup, et al. (2002) [58]	Adult Male Wistar Rats (Exercise Group *n* = 6 vs. Sedentary Sedentary Control Group *n* = 6)	12	western blot	post-intervention	4 weeks	treadmill exercise (5 days/week)	n.d.	Training enhanced expression of BDNF in the spinal gray matter fibers and, to a lesser extent, caused an increase of BDNF within neuronal perikarya. Immunocytochemical results show that long-lasting, moderate locomotor training, enhanced BDNF expression.
Fahimi, et al. (2017) [59]	Adult Male C57BL/6 Mice (Exercise Group *n* = 6 vs. Non-Exercise Control Group *n* = 6)	12	ELISA	post-intervention	5 weeks	treadmill running (5–7 days/week)	n.d.	BDNF significantly increased in mRNA levels following long-term exercise. A significant increase in BDNF levels in the exercise group was observed compared with the non-exercise control group.
Seifart, et al. (2010) [47]	Mice (Exercise Training Group *n* = 8/Control Group *n* = 8)	16	fluorescence-based real-time PCR	post-intervention	5 weeks	treadmill running (5 days/week)	n.d.	The BDNF mRNA levels in the hippocampus were higher in the trained mice than in the untrained mice from the control group. In the cerebral cortex, the BDNF mRNA levels were not significantly elevated by training but were comparable to those in the hippocampus of the untrained mice.
da Silva, et al. (2012) [60]	Male Wistar Rats (Exercise Group *n* = 27 vs. Control Group *n* = 27)	54	ELISA	post-intervention	±5–6 weeks	treadmill (39 sessions in total)	n.d.	A significant increase in hippocampal BDNF expression was noted in the exercise group (aerobic exercise program) when compared with the control group.
Alomari, et al. (2013) [61]	Male Wistar Rats (Forced Exercise Group *n* = 14–15 vs. Control Group *n* = 14–15)	28–30	ELISA	post-intervention	6 weeks	swimming (5 days/week)	n.d.	BDNF levels in the hippocampus were greater in the forced exercise training group when compared to the control group. BDNF was significantly higher in the forced exercise than in the control group.
Jin, et al. (2017) [62]	Adult Male Sprague-Dawley Rats (Young-Age Exercise Group *n* = 10 vs. Young-Age Control Group *n* = 10 vs. Adult-Age Exercise Group *n* = 10 vs. Adult-Age Control Group *n* = 10)	40	western blot	post-intervention	6 weeks	treadmill running (7 days/week)	n.d.	Treadmill exercise increased BDNF expression in both young and adult-aged rats compared with the young and adult-aged control groups.
So, et al. (2017) [63]	C57BL/6 Female Mice (Moderate Exercise Group vs. Fatiguing Exercise Group vs. Sedentary Control Group)	n.d.	ELISA	post-intervention	6 weeks	treadmill running (7 days/week)	n.d.	The moderate exercise group had significantly higher hippocampal BDNF expression levels when compared with the fatiguing exercise group and with the sedentary control group. No significant difference was found between the fatiguing and control groups.
Uysal, et al. (2015) [64]	Wistar Albino Rats (Involuntary Exercise Group *n* = 14 vs. Control Group *n* = 14)	28	ELISA	post-intervention	6 weeks	treadmill running (5 days/week)	n.d.	Prefrontal Cortex BDNF levels were greater in the males of the forced exercise group than in the control group. The BDNF levels were greater in females of the forced exercise group than in controls. Hippocampal BDNF levels were increased in the forced involuntary exercise group compared with the sedentary control group.
Cassilhas, et al. (2012) [65]	Adult Male Wistar Rats (Aerobic Exercise Group *n* = 11 vs. Sham Group *n* = 11 vs. Control Group *n* = 11)	33	ELISA	post-intervention	8 weeks	treadmill running (5 days/week)	n.d.	A significantly higher level of hippocampal BDNF was observed in the Aerobic Exercise Group compared with the Control Group. Aerobic Exercise modulates BDNF.
Costa, et al. (2012) [66]	Young Adult and Middle-Aged Male Wistar Rats (Young Adult Treadmill Running 1 days/week vs. Young Adult Treadmill Running 3 days/week vs. Young-Adult Treadmill Running 7 days/week) vs. Middle-Aged Treadmill Running 1 days/week vs. Middle-Aged Treadmill Running 3 days/week vs. Middle-Aged Treadmill Running 7 days/week vs. Young Adult Control Group vs. Middle-Aged Control Group)	n.d.	western blot	post-intervention	8 weeks	treadmill running (1/3/5 days/week)	60–75% VO2max	While a 1 day/week exercise program caused an increase in both proBDNF and BDNF in young rats, the other frequencies of exercise increased only BDNF levels when compared with the control group. The middle-aged exercise groups presented an increase in BDNF levels when compared with the control group.
Etemad, et al. (2014) [67]	Male Wistar Rats (2-Week-ShortTerm-Diabetic Exercise-Group *n* = 10 vs. 8-Week-LongTerm-Exercise-Diabetic Group *n* = 10 vs. Sedentary Diabetic Group *n* = 10 vs. Control Group *n* = 10)	40	ELISA	post-intervention	2 and 8 weeks	treadmill running (7 days/week)	n.d.	No significant change on BDNF levels was observed following a short and long-term aerobic exercise program compared with control group. There was also no significant difference between groups with 2 and 8 weeks of treadmill running in BDNF levels. An exercise protocol did not change significantly BDNF levels in either group.
Jiménez-Maldonado, et al. (2015) [68]	Male Wistar Rats (Moderate-Intensity Exercise Group *n* = 6 vs. High-Intensity Exercise Group *n* = 4 vs. Moderate-Intensity Exercise Group + TrkB Inhibitor Injections *n* = 4 vs. High-Intensity Exercise Group + TrkB Inhibitor Injections *n* = 5 vs. Sedentary Control Group *n* = 6)	25	ELISA	post-intervention	8 weeks	treadmill running (3 days/week)	moderate- or high-intensity (60 or 80% of VO2 max)	Chronic treadmill exercise significantly increased plasma BDNF. After training, plasma BDNF concentrations were significantly higher in the trained groups than those in the sedentary control. These results indicate that the circulating BDNF concentrations increased with chronic physical exercise.
Radak, et al. (2006) [69]	Wistar Rats (Exercise Trained *n* = 7 vs. Control *n* = 7)	14	ELISA	post-intervention at week 8	8 weeks	swimming training (5 days/week)	n.d.	Regular training significantly increases the production of BDNF. Exercise training significantly increased the protein content of BDNF, compared to control animal results after an 8-week aerobic exercise protocol.
Sheikhzaden, et al. (2015) [70]	Male Wistar Rats (2-week Exercise Group *n* = 10 vs. 8-week Exercise Group *n* = 10 vs. Control Group *n* = 10)	30	ELISA	post-intervention	2 and 8 weeks	treadmill running (7 days/week)	n.d.	Exercise did not change BDNF content in the hippocampus of trained animals. Neither long-term (8 weeks) nor short-term (2 weeks) exercise made any significant change to BDNF levels in the hippocampus.
Vilela, et al. (2017) [71]	Aging Wistar Rats (Aerobic Training Group vs. Untrained Control Group)	18	western blot	post-intervention	8 weeks	treadmill running (3–4 days/week)	n.d.	BDNF levels were increased after training for the aerobic exercise group compared to the rats in the untrained control group. The results show BDNF levels increased after aerobic training
*>12 weeks*								
Pietrelli, et al. (2018) [72]	Weaning Male Wistar Rats (Aerobically Exercised (AE) Group *n* = 30 vs. Sedentary Control Group *n* = 30)	60	ELISA	at 32-weeks and post-intervention	64 weeks	treadmill running (3 days/week)	low-moderate intensity: 60–70% VO2max	AE increased the BDNF concentration in most of the brain regions studied, nonetheless this response was modulated by age (significant differences due to exercise in the striatum were only seen among the old rats).

**Table 3:** Summary of studies that have reviewed the relationship between a chronic aerobic exercise intervention and the variation of NT-4/5 blood concentration levels in animal models.

Author(s)	Population Studied (Exercise Group *n* vs. Control Group *n*)	*n*	NT-4/5 Measure (Analysis Technique)	Time of Measure	Duration of Exercise	Aerobic Exercise (Days p/Week of Training)	Intensity of Exercise for Exercise Group (Physiological Parameter)	Main Outcomes
Maejima, et al. (2018) [18]	Senescence-Accelerated Mice (Resistant Sedentary Control Group *n* = 6 vs. Resistant Exercise Group *n* = 6 vs. Prone Sedentary Control Group *n* = 8 vs. Prone Exercise Group *n*= 7)	27	quantitative pcr based on real-time pcr	post-intervention	4 weeks	treadmill (5 days/week)	n.d.	Although exercise upregulated transcriptional NT-4 only in SAMP, NT-4 protein did not increase in SAMP or SAMR1. Two-way ANOVA for NT-4 expression showed no significant factorial effect.
Skup, et al. (2002) [58]	Adult Male Wistar Rats (Exercise Group *n* = 6 vs. Sedentary Control Group *n* = 6)	12	western blot	post-intervention	4 weeks	treadmill (5 days/week)	n.d.	Immunocytochemical results show that long-lasting, moderate training, enhanced NT-4/5 expression.

## Data Availability

Data used during the current study are available from the corresponding author on reasonable request.
